# Successful removal of B3 bile duct stones from B2 bile duct endoscopic ultrasound-guided hepaticogastrostomy route using wire-loop and bridge technique

**DOI:** 10.1055/a-2603-7425

**Published:** 2025-07-15

**Authors:** Hiroaki Tsuji, Ryota Sagami, Takao Sato, Hidefumi Nishikiori, Yasuhisa Hiroshima, Kazuhiro Mizukami, Kazunari Murakami

**Affiliations:** 1 157533 Department of Gastroenterology, Oita San-ai Medical Center, Oita, Japan; 2 13235 Department of Gastroenterology, Faculty of Medicine, Oita University, Oita, Japan

Endoscopic ultrasound-guided hepaticogastrostomy (EUS-HGS) is an effective drainage technique for difficult-to-access cases by usual endoscopic retrograde cholangiopancreatography (ERCP) 1 . Technical tips and reviews have been shown 2 3 , however, difficult situations exist.

A 36-year-old woman underwent extrahepatic bile duct resection and hepaticojejunostomy for congenital biliary duct dilatation at 3 years old. Percutaneous transhepatic biliary drainage (PTBD) and balloon enteroscope-assisted ERCP (BE-ERCP) were performed several times for repeated cholangitis. However, cholangitis caused by intrahepatic bile duct stones (IBDS) recurred. Computed tomography revealed IBDS in the B3 and B2 bile ducts ( Fig. 1 ). Repeated BE-ERCP was technically challenging/invasive because of the severe adhesion ( Fig. 2 **a** ).

**Fig. 1:**
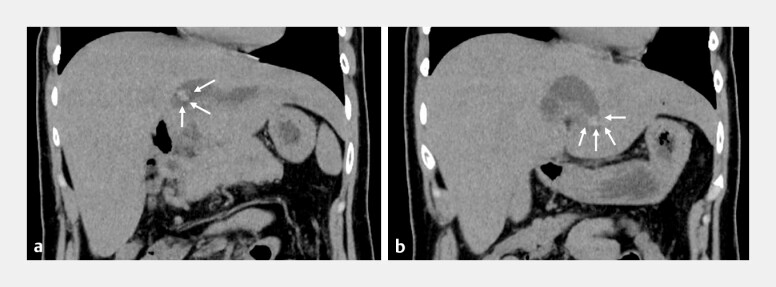
Imaging of computed tomography. **a** The left intrahepatic bile duct dilation and intrahepatic bile duct stones in the B2 branch. **b** The intrahepatic bile duct stone was also found in the B3 branch.

**Fig. 2:**
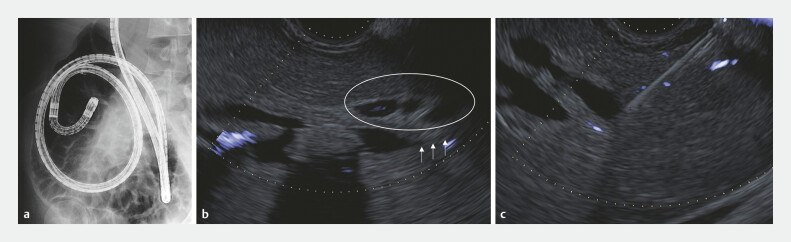
Imaging of BE-ERCP and EUS. **a** Severe adhesion would be expected from past BE-ERCP imaging. **b** No punctured route from the B3 bile duct because of no bile duct downstream dilation (white arrows) and many intervening vessels (white circle). **c** EUS-HGS was conducted from the B2 bile duct. Abbreviations: BE-ERCP, balloon enteroscope-assisted ERCP; EUS, endoscopic ultrasound; EUS-HGS, endoscopic ultrasound-guided hepaticogastrostomy.

First, EUS-HGS was conducted using a dedicated plastic stent (Through & Pass Type-IT; Gadelius Medical) from the B2 bile duct (5 mm) because there is no puncturing route from B3 with no bile duct downstream dilation and many intervening vessels ( Fig. 2 **b, c** ). Two weeks after the initial drainage, a dumbbell-shaped covered metal stent (M-Intraductal; Medicos Hirata) was deployed to dilate ESCR (EUS-guided created route). One week later, B3 IBDS removal was planned from the ESCR; however, the insertion of the basket catheter into the B3 was difficult because of the sharp angle between B2 and B3 bile ducts.

A 0.025-inch soft guide wire (TRU wire; Medicos Hirata) passed from the B2 EUS-HGS route to B3 using the wire-loop and bridge technique ( Video 1 ).

Making a loop of a guidewire and swinging the EUS scope could be useful for passing the wire between the sharp angles of B2 and B3 bile ducts and stone removal.Video 1

After the balloon catheter (Extraction, Xemex, Zeon Medical) was inserted, B3 IBDSs could be moved to the B2 bile duct without dislocating the wire ( Fig. 3 **b** ). Finally, all IBDSs were removed from the ESCR ( Fig. 3 **c, d** ).

**Fig. 3:**
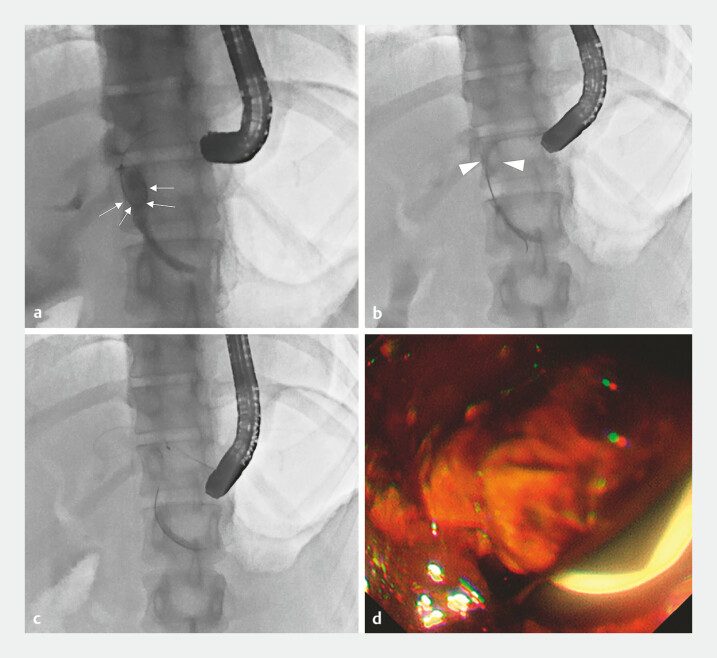
The X-ray fluorescence and endoscopic image of endoscopic stone removal. **a** X-ray image of wire manipulation using wire-loop and bridge technique (white arrows). **b** X-ray image of B3 stone movement to B2 bile duct by a balloon catheter (white arrow heads). **c** X-ray image of stone removal by the basket catheter. **d** Endoscopic image of the removal of stones from the ESCR. Abbreviation: ESCR, EUS-guided created route.

This guidewire manipulation technique with a balloon catheter may help remove IBDSs from the other EUS-HGS route for whom the EUS-puncturing route is restricted.

Endoscopy_UCTN_Code_TTT_1AS_2AH

## Notes

Endoscopy E-Videos https://eref.thieme.de/e-videos
*E-Videos* is an open access online section of the journal *Endoscopy* , reporting on interesting cases and new techniques in gastroenterological endoscopy. All papers include a high-quality video and are published with a Creative Commons CC-BY license. Endoscopy E-Videos qualify for HINARI discounts and waivers and eligibility is automatically checked during the submission process. We grant 100% waivers to articles whose corresponding authors are based in Group A countries and 50% waivers to those who are based in Group B countries as classified by Research4Life (see: https://www.research4life.org/access/eligibility/ ). This section has its own submission website at https://mc.manuscriptcentral.com/e-videos .
